# Abernethy Malformation Type 1b

**DOI:** 10.5334/jbsr.2431

**Published:** 2021-04-06

**Authors:** Evelien Claesen, Steven Van den Berge, Enrique Havinga

**Affiliations:** 1 UZ Leuven, BE

**Keywords:** Congenital extra-hepatic portosystemic shunt, abernethy malformation, pediatrics, abdominal ultrasound, abdominal CT

## Abstract

**Teaching point:** Extrahepatic portosystemic shunt is a rare congenital condition, associated with other congenital anomalies, for which prompt radiological diagnosis and treatment are important to prevent complications.

## Case Report

A four-month-old female patient with prenatal diagnosis of Tetralogy of Fallot underwent abdominal ultrasound for rising bilirubin levels after cardiosurgery.

It revealed homogeneous liver parenchyma, normal gallbladder, and no dilatation of the biliary ducts. However, the portal vein could not be demonstrated. Where we would normally expect the intra-hepatic portal vein there was a hyperechoic smooth band of 3–4 mm (***Figure A***, arrow). No portal vein Doppler signal could be detected in the liver. Pulsed-wave Doppler in the hepatic and splenic veins showed the same flow pattern (***Figure B***), indicating a communication between the systemic and splanchnic veins. A contrast-enhanced computed tomography (CT) of the abdomen was performed to demonstrate properly the anatomy of the splanchnic veins. It confirmed a venous malformation, with confluence of the splenic and superior mesenteric vein into a common trunk that drained directly into the right atrium, also named “Abernethy malformation type 1b” (***Figure C***, coronal maximum intensity projection, red arrow: splenic vein, black arrow: superior mesenteric vein, white arrow: shunt, star: right atrium). The patient eventually died a month after surgery without being treated for the malformation.

**Figure 1:**
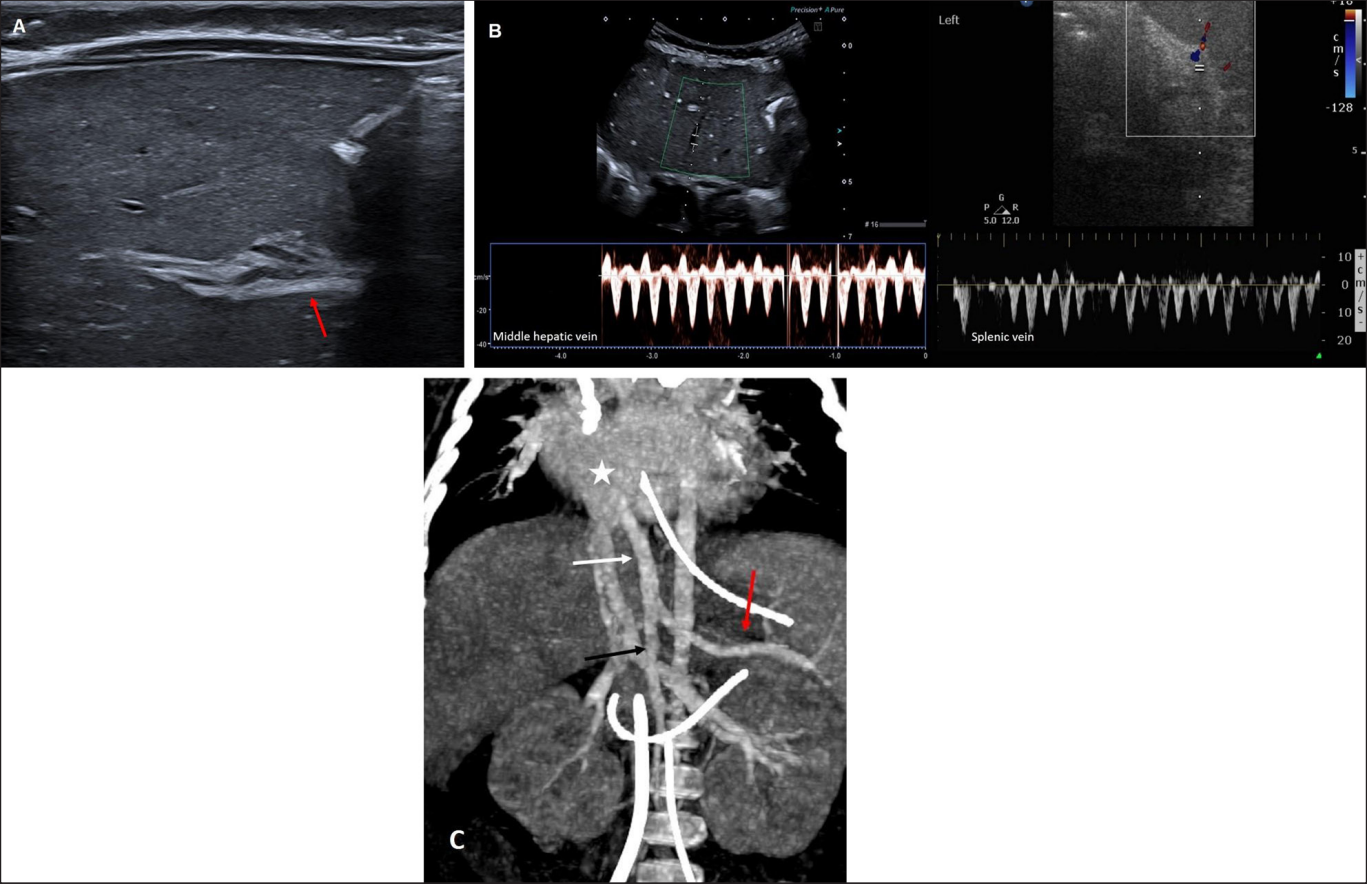
A, B and C.

## Comment

A congenital extra-hepatic portosystemic shunt (CEPS) is a rare malformation in which the portomesenteric blood drains directly into the systemic circulation. CEPS is classified into two types. In type 1 malformations there is a congenital absence of the portal vein. These are further divided into those in which the splenic and superior mesenteric vein drain separately into a systemic vein (type 1a) and those in which the two veins form a common trunk that drains into the systemic circulation (type 1b). In contrast, the portal vein is preserved in type 2 malformation, but some of the portal flow is bypassed to the systemic circulation through a side-to-side shunt [1].

Clinical manifestations are related to abnormal hepatic development, the portosystemic shunt and associated congenital anomalies, for instance congenital heart defects. The presence and extent of complications, such as portosystemic encephalopathy, and associated anomalies determines the prognosis [1].

Usually, CEPS is discovered incidentally on routine ultrasound (US) or in a symptomatic child with nonspecific liver disease. Absence of the portal vein is most often demonstrated. In contrast to other causes of spontaneous shunts, patients with CEPS do not have imaging features of portal hypertension [1]. CT angiography is used for further classification of the shunt, because it can clearly depict the course of the portosystemic shunt and identify portal branches.

It is essential to determine the type of shunt because treatment options are totally different [1]. Eventually, the therapeutic approach in patients with type 1 malformations is liver transplantation, whereas shunt occlusion is an effective treatment for patients with type 2 malformations.
